# Challenges for the human immune system after leaving Earth

**DOI:** 10.1038/s41526-024-00446-9

**Published:** 2024-11-18

**Authors:** Shannon Marchal, Alexander Choukér, Jürgen Bereiter-Hahn, Armin Kraus, Daniela Grimm, Marcus Krüger

**Affiliations:** 1 Department of Microgravity and Translational Regenerative Medicine, Otto-von-Guericke University, Universitätsplatz 2, Magdeburg, Germany; 2 Laboratory of Translational Research “Stress and Immunity”, Department of Anesthesiology, LMU University Hospital, LMU Munich, Marchioninistr. 15, Munich, Germany; 3 Institute for Cell Biology and Neurosciences, Goethe University Frankfurt, Frankfurt am Main, Germany; 4 Clinic for Plastic, Aesthetic and Hand Surgery, University Hospital Magdeburg, Magdeburg, Germany; 5 Research Group “Magdeburger Arbeitsgemeinschaft für Forschung unter Raumfahrt- und Schwerelosigkeitsbedingungen” (MARS), Otto-von-Guericke University, Universitätsplatz 2, Magdeburg, Germany; 6 Department of Biomedicine, Aarhus University, Aarhus, Denmark

**Keywords:** Molecular medicine, Immunology, Environmental sciences

## Abstract

From the start of life on Earth, several immune defense mechanisms have evolved to guarantee cellular integrity, homeostasis, and host survival. All these sophisticated balances as shaped by and towards the environmental needs have occurred over hundreds of millions of years. Human spaceflight involves various health hazards, such as higher levels of radiation, altered gravity, isolation and confinement, living in tight quarters, and stress associated with being away from home. A growing body of evidence points towards immunological changes in astronauts, including heightened pro-inflammatory responses, reactivation of latent viruses, and cell-mediated alterations, reflecting a dysbalanced state in astronauts. Simultaneously, enhanced pathogenicity, virulence, and drug resistance properties of microorganisms tip the scale out of favor for prolonged stay in space. As we have learned from the past, we see potential for the human immune system, forged and maintained throughout evolutionary history, to adapt to the space exposome. It is unlikely that this will happen in the short time frames set for current space exploration missions. Instead, major risks to astronaut health need to be addressed first, before humans can safely evolve into the space environment.

## Introduction

**Fig. 1:**
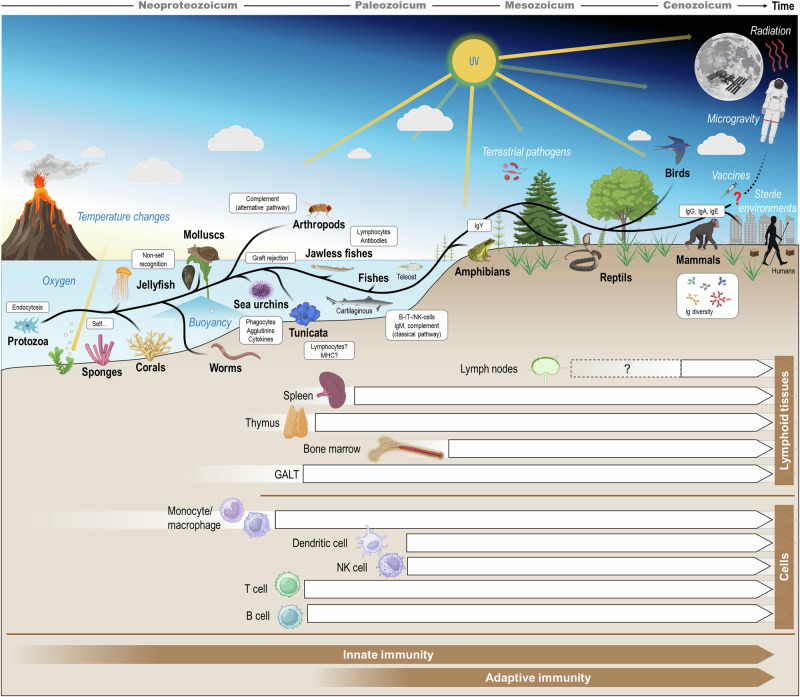
The immune system of animals on Earth has evolved over a period of one billion years. New developments are listed in the white boxes, new challenges are written in italics. The occurrence of important components of the human immune system is shown below the development history. Innate immunity is the oldest form of defense and occurs to some degree in all species and comprises of physical barriers, chemical products and components (e.g., acids, enzymes, peptides), and immune cells. The adaptive immunity (humoral compounds, B and T cells) emerged around 600-450 million years ago in vertebrates. Abbreviations: GALT gut-associated lymphoid tissue, Ig immunoglobulin, MHC major histocompatibility complex, NK natural killer (cell).

Since the appearance of the first eukaryotic cells at least 2.7 billion years ago, several defense mechanisms have evolved to ensure cellular integrity, homeostasis, and host survival (Fig. 1). It has become textbook knowledge that the human immune defense operates through two vital, interconnected avenues: the innate immunity and adaptive immunity. The roots of innate immune mechanisms trace back almost to the dawn of life itself, evolving alongside single-celled organisms over billions of years.

Host response to invading pathogens has become a basic physiological response of all living organisms^1^. Even unicellular invertebrates possess cellular receptors that bind to foreign elements and distinguish the self from the foreign. As life diversified into multicellular organisms, the complexity of both organisms and pathogens increased, prompting a diverse array of innate defense mechanisms^2^. In multicellular invertebrates, this ability is associated with the presence of specialized phagocytes. These cells have a macrophage-like appearance and a similar function, which is prominent even at the earliest evolutionary stage. Well-conserved pathogen recognition receptors (such as Scavenger receptors, Toll-like receptors, or Nod-like receptors) on the cell surface recognize typical molecular patterns expressed by various pathogens (e.g., bacteria, viruses, fungi, protozoa, helminths) through receptor-ligand binding and initiate a complex cascade of cellular reactions which lead to the production of effector molecules^3–5^. Cytokines, even in lower invertebrates, are involved in this orchestration of responses that can ultimately lead to the elimination or inactivation of the invader^6,7^. While insects such as *Drosophila* rely on the innate immune system^8^, spiders and crabs have an alternative complement pathway^9^. Echinoderms show numerous variants of innate recognition and effector molecules that enable rapid and innate responses to various pathogens despite their lack of adaptive responses (Fig. 1)^10^. The high specificity, maturation of antibodies, immunological memory, and secondary responses of adaptive immunity were so successful that higher vertebrates were able to reduce the variants of innate molecules originating from invertebrates and lower vertebrates^3^. Nevertheless, vertebrates link the two arms in an intricate, interdependent network^11^.

To survive, organisms at all evolutionary stages have used available genes and functions, some of which have been lost or have changed function during time. The molecular mechanisms involved in the evolution of immune molecules can be as diverse as gene duplication, deletions, alternative splicing, gene combination, domain displacement, retrotransposition, and gene conversion, in addition to simple base substitutions. Variable regulation of gene expression may also have played a role. However, the evolution of immunity is not limited to the temporo-spatial evolution of entire biocenoses; variations in pathogens and individuals over the lifetime of a host species or changes in the frequency of lymphocyte clones within an individual during a single infection also contribute to adaption. In general, all living systems have the capacity of perceiving their environments and to hereby increase survivability momentarily and over many generation cycles. But does that work when we are more “suddenly”—in the scale of our planet’s history—leave our habitat Earth, where we have co-evolved for thousands of years?

It is now known that the interaction of cells changes significantly under the conditions of spaceflight^12–14^, affecting differentiation, the mutual influence of tissues during differentiation, and also immune responses, the first steps of which consist of cell-cell interactions (host response). Based on these very general responses to an evolutionarily ubiquitous situation, it can be assumed that the immune system is affected under the unique conditions of spaceflight. This review aims to combine our knowledge of our immune system’s adaptability and the effects of the space environment on astronauts’ immune systems to predict how our immunity might change after leaving Earth and what challenges, threats, but also opportunities might arise.

## The human immune system in space

The human immune system consists of two branches and many components. The innate immune system builds up the “first line of defense”, consisting of elements such as mechanical barriers (skin and mucous layer), followed by neutrophils, macrophages, monocytes, acute phase proteins, cytokines, and the complement system on the cellular and molecular level^15^. A fundamental characteristic of the adaptive immune system is the ability to distinguish what is “self” and what is “non-self”. Missing foreign pathogens may lead to infection, missing mutated own cells may lead to tumor formation, but over-aggression of the immune system aimed at its own organism may lead to autoimmune diseases. Careful balance of this system is vital for the avoidance of being overwhelmed by infection, counteract tumor formation, but on the other hand not to attack the self^16^. The unique conditions of the space exposome (Box 1), as they had never been experienced before during evolution, present a great challenge to keep this sophisticated scale balanced.

**Fig. 2:**
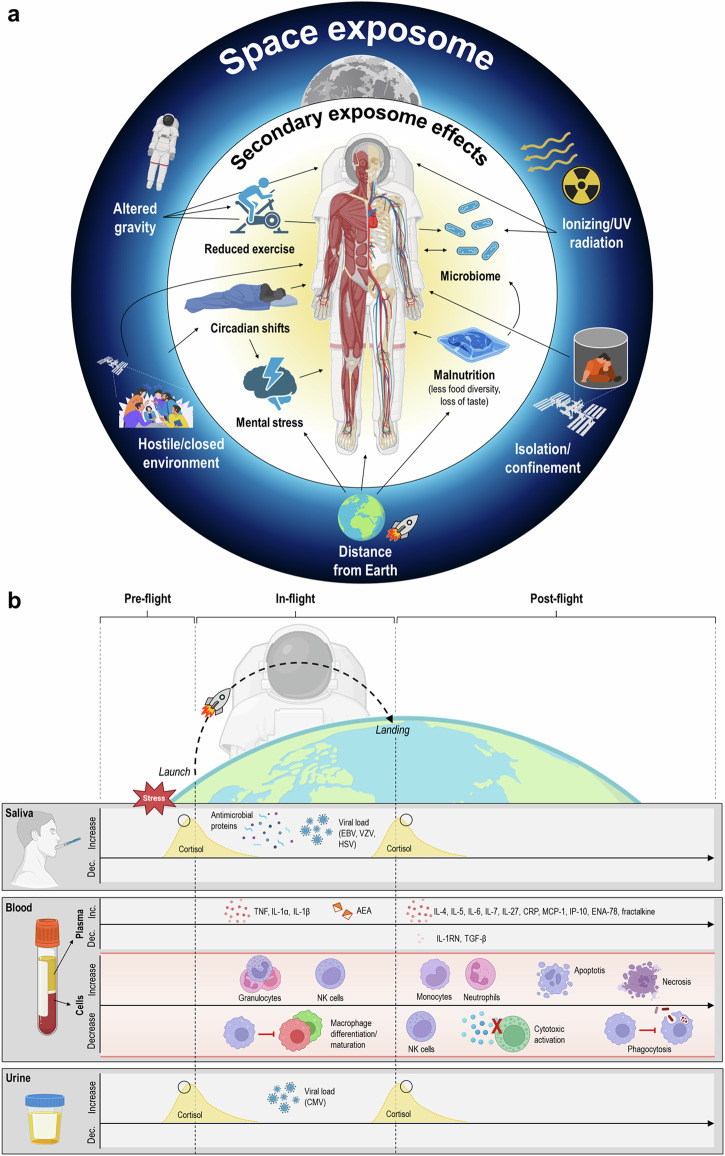
Influence of space travel on the human body. **a** The “space exposome”: environmental factors in space can have a direct or indirect effect (secondary exposome effects) on the health of astronauts. **b** Immunological changes in astronauts before, during, and after a space mission. The black circles mark a snapshot of the presumed cortisone level. Abbreviations: AEA blood anandamide, CMV cytomegalovirus, EBV Epstein-Barr virus, HSV herpes simplex virus, IL interleukin, NK natural killer (cell), TGF transforming growth factor, TNF tumor necrosis factor, VZV varicella zoster virus.

Box 1 The space exposome A few years ago, the “exposome” was proposed as a new paradigm encompassing the totality of environmental (non-genetic) influences on the human body that complement the genome. The main health risks of spaceflight include higher levels of harmful radiation^156^, altered gravity^157^, long periods of isolation and confinement^158^, a closed and potentially hostile living environment^159^, and the stress associated with being away from home (communication delays, autonomous medical care, etc.)^160^. However, secondary effects such as reduced exercise leading to microgravity-related movement problems^161^, unbalanced nutrient intake due to reduced food diversity, and potential impairment of the sense of taste^162^, as well as disruption of the circadian clock^163,164^, also contribute to astronaut health issues. Crewmembers do not experience these stressors independently, so it is important to also consider their combined effects on human physiology and performance. This “space exposome” (Fig. 2a), in conjunction with individual genetics, can determine the effects of spaceflight on the human immune system^151,165^.

### Inflammatory response

Spaceflight-associated immune dysfunction has long been recognized by medical professionals. Today, a growing body of evidence points towards an increased inflammatory state in astronauts observed both during spaceflight and on return to Earth (RTE) (Fig. 2b)^17–21^. Key inflammatory cytokines released during early immune responses to infections are TNF, IL-1, and IL-6. These cytokines are critical for initiating cell recruitment and local inflammation, essential in the clearance of many pathogens^22^. Increased plasma concentrations of TNF, IL-1α, and IL-1β have been observed in astronauts who have flown in space^17–19^. Immediately on RTE, astronauts showed a significant spike in IL-6 plasma concentrations^19^, together with other pro-inflammatory cytokines (IL-10, CRP, MCP-1, IL-27), myokines (IL-4, IL-5, IL-7) and chemokines (interferon gamma-induced protein 10, ENA-78, fractalkine) (Fig. 2b). Kim et al. recently hypothesized the source of these immune makers to originate from the muscle and other tissues during exercise, indicating a physiological response to microgravity rather than a solely inflammatory response^23^. Simultaneously, high inflight levels of regulatory cytokines IL-10, IL-1 receptor antagonist protein (IL-1RN), and transforming growth factor β (TGF-β) dropped on RTE, suggesting a pro-inflammatory immune status with a concomitant reduction in the anti-inflammatory capacity^21^. In line with these findings, a one-year space mission revealed increased levels of lysophospholipids containing pro-inflammatory omega-6 20:4 fatty acid, together with a decrease in lysophospholipids containing anti-inflammatory omega-3 20:5 fatty acid^20^. The ability of host defense to rapidly identify and eradicate foreign microbes and activate pro-inflammatory pathways relies heavily on antimicrobial proteins (AMPs) to amplify protection through biochemical mechanisms^24^. Findings demonstrated elevated levels of AMPs, such as salivary IgA (sIgA), lysozyme, and LL-37 during spaceflight^25^.

### Stress response

Life in space is characterized by unique, but stressful conditions (see “space exposome”, Box 1)^26^. Psychological stress has been linked to various immunological processes, including inflammatory processes, wound healing, responses to infectious agents, vaccination effects, and pathogenesis (autoimmunity, cancer)^27,28^. Spaceflight-associated stressors could chronically amplify the release of stress hormones, which in turn could negatively affect the human immune system (Fig. 2b). Interestingly, the classical stress hormones (cortisol and catecholamines) evaluated during spaceflight did not significantly differ between daytime- and mission time points^21,29,30^. A rise in salivary and urinary cortisol was observed in the early inflight phase and upon landing but returned to baseline values across the 6-month mission duration and within 30 days after landing^29–31^. Interestingly, non-classical markers such as plasma anandamide (AEA) were increased during flight compared to control subjects, reflecting a general activation of stress response systems^21^. On a cellular level, altered gravity (microgravity) and radiation cause metabolic stress. Consequently, production and accumulation of excessive reactive oxygen species (ROS) cause oxidative stress that can harm lipids, proteins, carbohydrates, and DNA across all organ systems^32^. ROS have various functional roles in immunological signaling. They are key players in the migration and activation of polymorphonuclear leukocytes to the site of injury. Impaired ROS production hinders phagocytic function of neutrophils and macrophages^33^. In the adaptive immune system, ROS plays a critical role in T cell signaling and T cell activation^34^. Astronauts in space have shown a dysregulation of CD8^+^ T cell infiltration in ganglions, permitting the reactivation and/or shedding of latent viruses^35,36^. Immunological changes in astronauts are evidenced by the reactivation of latent human herpes viruses (HHV)^37^. Latent virus reactivations have been observed in astronauts during both short-duration shuttle flights (10–16 days) and long-duration ISS flights (≥180 days). Following reactivation, viruses are shed in the body fluids of astronauts, such as saliva, plasma, and urine^30,37^. Around 60% (14 out of 23) of all astronauts from long-duration ISS missions shed at least one or more HHV in their saliva or urine. Epstein-Barr virus (EBV) and varicella zoster virus (VZV) were detected in the saliva of approximately 65% and 96% of all astronauts, respectively. Cytomegalovirus (CMV) was detected in the urine of approximately 61% of all astronauts. Magnitude and frequency of viral shedding increased with mission duration^30^. Interestingly, higher concentrations of salivary cortisol levels were observed in astronauts who shed a latent herpes virus compared to those who did not shed^25^. Spielmann demonstrated elevated levels of plasma AMPs (lysozyme and human neutrophil peptide, HNP1-3) in astronauts who exhibited EBV and VZV reactivations during flight. Plasma concentrations of LL-37 decreased upon return to Earth and were associated with greater CMV reactivation^38^. A single case study reported persistent dermatitis (herpes simplex virus 1, HSV-1) during flight, demonstrating elevated stress markers, circulating inflammatory cytokines, and HSV-1 DNA levels in saliva and lesion swab^39^. These findings suggest a role for stress in the reactivation of viral infections during spaceflight^40^.

### Cell-mediated immunity

Cell-mediated immunity relies on lymphoid homeostasis, important for the regulation of immune responses (Fig. 2b). Both spaceflight and ground-based experiments have shown inhibition of macrophage differentiation from mouse hematopoietic stem cells^41^. Differentiation and maturation of lymphocytes from the bone marrow may be influenced by the effect of weightlessness on the human skeletal system^42,43^. Astronauts’ skeletal health (bone mineral density) declines with a rate of 0.5–1.5% per month spent in space^44^. Some level of overlap has been observed between astronauts’ immune systems and participants exposed to prolonged bed rest, a common analog for human microgravity studies on Earth. EBV reactivations were observed in subjects exposed to a 60-day bed rest study, suggesting an immunocompromised state^45,46^. Inflammatory cytokines, including IL-1, IL-6, and TNF, were found to have a significant effect on the bone remodeling process, mostly driving the system in the direction of resorption^47^.

Peripheral leukocyte distribution varies between inflight and post-flight measurements. Relative to preflight values, the total number of leukocytes, granulocytes, and natural killer (NK) cells increased during spaceflight. Levels of lymphocytes, monocytes, lymphocyte subsets (B and T cells), and T cell subsets (CD4^+^ and CD8^+^) were unaltered^48,49^. On RTE, the total number of leukocytes remained elevated compared to preflight values. Neutrophil and monocyte count increased by 50%, shortly after landing^18,21,50,51^. On the other hand, NK cell count decreased upon RTE^18,21,52,53^. One study even reported a 60% drop in NK cells shortly after landing^21^. B cell homeostasis was maintained during long-duration spaceflight^46,49^, however, few studies have shown increased levels of B cells on RTE^18,21^. Findings on immune responses immediately after spaceflight may be confounded by the high-*g* reentry and stressors related to re-adaptation to terrestrial gravity following prolonged spaceflight missions.

Following short-duration spaceflight missions (5-11 days), monocytes displayed a reduced ability to engulf *E. coli*, elicited an oxidative burst, and degranulated^54^. Phagocytosis and oxidative burst capacities in neutrophils were significantly lower post-flight after a 9-11-day mission but not after a 5-day mission^50^. Lipopolysaccharide stimulation of astronauts’ monocytes produced reduced amounts of pro-inflammatory cytokines IL-6 and IL-1β and higher amounts of anti-inflammatory cytokine IL-1RN compared to controls^55^. Findings suggest that monocyte and neutrophil function may be affected by factors associated with spaceflight, shown by a reduced responsiveness of host defense cells against invading pathogens. NK cell cytotoxic activation against K562 leukemia targets was reduced by 50% in astronauts during spaceflight compared to ground controls^56^. Exposure to microgravity conditions increased their apoptotic and necrotic activity concomitantly with delayed hypersensitivity responses^57^. B cell homeostasis was supported by unchanged plasma levels of immunoglobulin-free light chains, IgG, and IgM, during long-duration spaceflight. All except for IgA levels, that increased during spaceflight^49^. Consistently with animal models, spaceflight did not affect the immunoglobulin repertoires of mice after short-duration spaceflight^58^. Cell number and cell function determine immunological responses first and foremost. The current findings demonstrate important alterations in cell function, taken together with reactivation of latent viruses, suggesting an overall compromised immune response.

### Immune adaptations to human spaceflight

Astronauts re-exposed to the space environment have shown reduced immunological adaptations. Experienced astronauts had lower levels of AMPs (α-amylase, lysozyme, and LL-37) in their saliva and higher concentrations of sIgA compared to rookie astronauts, observed during and after spaceflight^25^. The decline in NK cell function, as described earlier, was more pronounced in rookie astronauts compared to their experienced counterparts^56^. Immune adaptation to the space environment is necessary for organisms’ safe travel away from Earth. These results show the immune’s capacity to learn from a previous exposure to the spaceflight environment. More re-exposure studies are required to examine the extent of immune adaptation and immunological memory in response to various space conditions.

### Life and oxygen in space

Whilst long-duration space missions induce complex orchestrated and multidirectional immune affecting stress responses as described, the life conditions in future extreme long-duration missions or on planetary colonies are of special interest. Lowering the partial pressure of oxygen in the spaceship on a long interplanetary journey or on-site in the habitat is likely to be equivalent or lower to an oxygen concentration of 19%^59^. Oxygen content may be even lower as for technical reasons, fire hazards or to reduce radiation effects aggravated by oxygen. The effects of lowered oxygen availability on the human immune system can be significant. As paralleling the evolution of the immune system, life has become an “oxygenated” life from the Cambrian explosion onwards and evolved over several millions of years in a dynamic state of increasing oxygen tension to an Earth's atmosphere as of today. Interestingly the mitochondrial genome in eukaryotes retains similarity to its prokaryotic ancestor. Mitochondrial genes that have been conserved across the evolution include ribosomal (rRNA) and transfer RNA (tRNA) genes and a small number of genes that are related to the encoding of proteins involved in electron transport and ATP synthesis (i.e., the ATP synthase to synthesize ATP from ADP)^60^. The breakdown of ATP stands as the primary step for metabolic energy, also in eukaryotic cells, and is related to oxygen availability. This applies also within cells, as localized regions might encounter ATP scarcity due to increased local consumption or reduced ATP production during periods of hypoxia^61^.

Because ATP is not only a key mitochondrial metabolite and “currency” of energy but also a signal transmitter in the purinergic signaling, it plays a pivotal role in regulating diverse cellular processes such as tissue oxygen tension and mitochondrial action. For instance, when various mammalian cell types are stimulated, they release ATP and through several families of ectonucleotidases its degradation products. The ATP and the derived ligands can bind to various receptors and induce auto- and paracrine feedback^62–64^. In the last three decades, 19 distinct receptor subtypes of purinergic receptors were characterized capable of recognizing these ligands (eight P2Y subtypes, seven P2X subtypes, and four P1 (adenosine A1, A2A, A2B, and A3)). Depending on the ligand and receptor affinity and G-protein coupling, binding to the purinergic receptors can either enhance or inhibit the activation of immune cells. These processes do happen in parallel to enable balanced responses. Experimental and human research has shown that reduction of the oxygen tension can affect these pathways and affect ATP metabolism triggering purine-mediated immune modulation^63,65^. These effects are not only a function of the reduction of the oxygen tension but also a function of time of exposition since initial immune modulatory effects can be changed over time. Humans overwintering in the high-altitude Antarctic environment (Concordia Station, Dome C) characterized for its hypoxic conditions, observed a dynamic immune activation and a two-step escalation/activation pattern^66^.

The early phase was characterized by moderately sensitized global immune responses, while after several months, immune responses were highly upregulated. The cytokine responses to an ex vivo stimulation were markedly raised. The parallel quantitative polymerase chain reaction analyses from blood revealed that key elements of the purinergic system were significantly altered and dysregulated, indicating to some extent an adaptive process of disinhibition of purinergic signaling^66^. The dysregulation of the immune system seen during overwintering corresponded to the decreased expression of B and T lymphocyte attenuators (BTLA). Several studies demonstrated its critical role in up-regulation of inflammation and one of the first reports on BTLA expression in humans suffering from Behcet’s disease (auto-inflammatory vasculitis) to be associated with a diminished expression of BTLA^67^. The signaling lymphocytic activation molecule family 1 receptor (SLAMF‐1) was increased at the early phase of the Antarctic deployment and remained elevated. This receptor is considered to be related to the control of humoral autoimmunity, primarily via CD4^+^ T cells^68^. As such, alterations of SLAMF-1 and BTLA expression are significantly involved in hypersensitivity diseases and might be related to the increased incidence of clinically relevant hypersensitivity reactions, either allergic or autoimmune when exposed to such extreme conditions^66,69^. Moreover, the separate and combined effects of hypoxia and (simulated) reduced gravity may further modulate these deconditioning of vital physiological systems by additive increase of purines in humans^70^.

## Space environment and microbes

### Pathogenicity and virulence

**Fig. 3:**
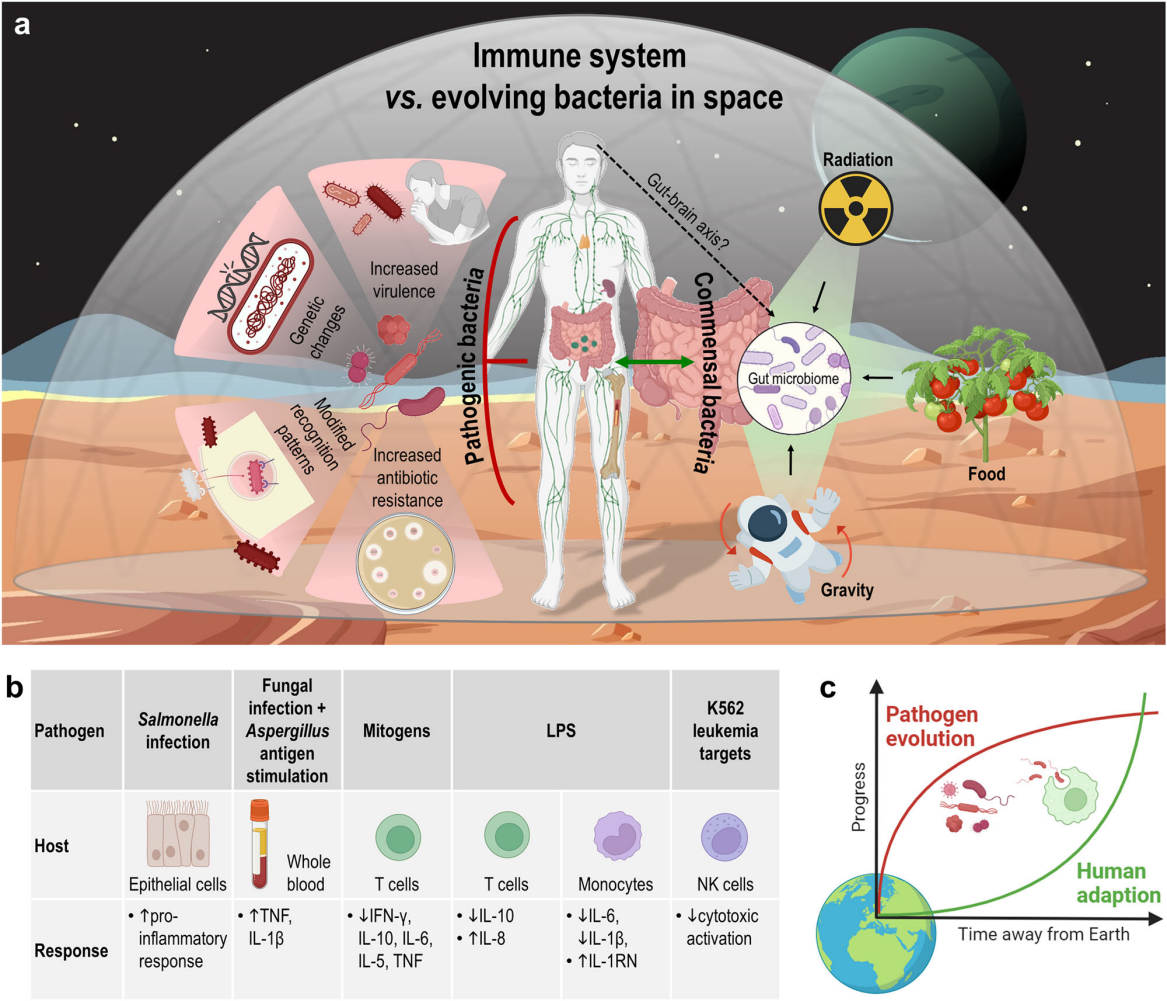
Friend or enemy?—Challenges for the human immune system due to bacteria in space. **a** The space environment influences both the biology of pathogens (the left part summarizes already described observations on pathogenic microorganisms), from which the immune system must protect astronauts, and the commensal bacterial flora of the body (right part). The human microbiome has to cope with the changed conditions in space (radiation, altered gravity, food supply, mental challenges), the effects of which have not yet been clarified in detail (**b**) Host-pathogen-associated cytokine profiles. **c** Rate of evolution for varying species: microorganisms and humans. (Abbreviations: IFN interferon, IL interleukin, LPS lipopolysaccharide, NK natural killer (cell), TNF tumor necrosis factor.

The ISS harbors a variety of microorganisms, including contaminants from Earth, components of experiments and the normal microbiota of crewmembers. Space modules provide exceptional conditions for Earth’s microbes to grow and spread due to high radiation doses, microgravity and enclosed, compact environments (i.e., controlled humidity, controlled temperature, O_2_/CO_2_ ratio and long exposure time)^71,72^. The human body’s microbiome is prone to external forces, including the ISS microbiome, as they are in constant exchange and interaction. The ISS microbiome is dominated by human-associated microbes, with *Streptococcus, Corynebacterium, Lactobacillus, Acinetobacter*, and *Staphylococcus* as dominant taxa. Microbiome composition aboard the ISS changes over time, shown by an increase in microbial diversity after two cargo deliveries^73^. The space environment induces key changes in microbial cells that are directly relevant to infectious disease (Fig. 3a). This includes alterations of microbial growth rates, antibiotic resistance, microbial invasion of host tissue, organism virulence (the microbes’ ability to cause disease) and genetic changes within the microbe^74–78^. For instance, *S*. Typhimurium and *E. coli* displayed increased growth and culture densities during spaceflight^75,79,80^. Both *S. aureus* and *E. coli* showed increased resistance to antibiotics^81^. Genetic changes include alterations in gene expression patterns and genetic transfer^82^. Other observations include biofilm formation in *Pseudomonas aeruginosa*, morphology changes such as thickening of the cell wall of *S. aureus*, and greater cell size of *Proteus vulgaris*^82–84^. Animal models display shorter survival times compared to controls when infected with *Serratia marcescens* in fruit flies and *A. fumigatus* in larval zebrafish^74^. Ground control experiments demonstrated an increased virulence of *S*. Typhimurium in a murine model^85^. *S*. Typhimurium and *E. coli* showed enhanced resistance against all kinds of stress (acid-, thermal-, and osmotic stress) under simulated microgravity conditions^77^. Microbial presence in biofilms shows more resistance to antibiotics and other stressors^86^. These microbial characteristics are of importance to astronaut health and the integrity of the spacecraft. Adaptation of terrestrial pathogens to “alien” environments could lead to modified microorganisms with different pathogenic potential. It is unknown how the human immune system might react to these modifications due to different metabolic and cellular structures. Immune recognition might fail or in contrast, overreact to these “alien” microbes^87^.

### Commensal bacteria

The human gut microbiome can affect immunological responses and thus impact the general health of astronauts during spaceflight. During both short- and long-duration spaceflight missions, changes in the gut-, nasal- and oral bacterial profiles of astronauts have been observed^88^. Historical findings reported an increased microbial count with reduced microbial diversity in astronauts’ stool samples^89,90^. The 1-year twin study showed decreased metabolites in the gut microbiome in space, such as 3-indole propionic acid, which has anti-inflammatory effects. The authors propose these changes are due to nutritional restraints related to spaceflight^20^. Changes in microbial metabolites, diversity loss, and interference in energy metabolisms are three recognized microbial disturbances that may adversely impact human health. There is robust evidence that a limited gut microbial diversity leads to a higher prevalence of chronic inflammatory conditions such as inflammatory bowel disease or obesity^91–93^.

The microbiota from nine astronauts who spent a year on the ISS showed a space-induced decrease in the population of three bacterial genera with anti-inflammatory properties: intestinal *Fusicatenibacter*, *Pseudobutyvibrio*, and *Akkermansia*. Interestingly, an abundance of *Bacteroides* with a decrease in *Lactobacillus* and *Bifidobacterium* was observed after short-duration spaceflight missions^94^. *Bacteroides* reproduce rapidly under stressful conditions and increase subsequently with a weakening of the immune system. The *Lactobacillus* and *Bifidobacterium* species may interfere with the functioning of the human immune system and gut microbiota, causing latent viral reactivations and increasing the number opportunistic pathogens in the gut^88^. Non-Western microbiomes consist of greater bacterial diversity compared to Western microbiomes^95–97^. The differences between populations could point to cultural and environmental factors. Diets with higher levels of fibers and lower amounts of sugar, fat, and meat, typical for non-Western diets, promote bacterial richness in the gut^98^. It is likely that other environmental factors, other than nutritional restraints, have an impact on the gut microbiome (Fig. 3a). Earth examples have shown that the effects of antibiotics on the bacterial communities result in biodiversity loss and compositional imbalance^99^. Reduced contact with “old friends” (bacteria and parasites common in the natural environment) increases the risk of developing asthma, allergies, or other hypersensitivity diseases^100,101^.

Similarly, the human skin microbiome is likely to show adaptations to the space environment. A 6-month mission to the ISS showed reduced diversity of the skin microbiome, observed in 10 astronauts. An increased colonization by *Malassezia*, a lipophilic skin fungus, was observed compared to preflight samples^102^. Similar observations were made in an astronaut during a 1-year stay on the ISS^103^. The authors associated the microbiome of an astronaut to those of patients with seborrheic dermatitis, a condition sensitive to stress and immunosuppression.

Furthermore, host-pathogen interaction during spaceflight and associated cytokine profiles would provide valuable information regarding immune response effectiveness (Fig. 3b). Human intestinal epithelial cells, exposed to an infection with *Salmonella* Typhimurium showed a heightened pro-inflammatory response compared to uninfected cells and matching ground controls. Consistent with the inflammatory response was the amplified induction of genes encoding pro-inflammatory mediators and wound healing^14^. Similar findings demonstrated an amplified response of TNF and IL-1β following fungal antigen stimulation and *Aspergillus* antigen stimulation of whole blood samples from returning astronauts^21^. Mitogen-stimulated T cells produced reduced levels of IFN-γ, IL-10, IL-6, IL-5, and TNF, persistent during spaceflight. Lipopolysaccharide-stimulated T cells produced reduced levels of IL-10, but increased levels of the neutrophil chemoattractant factor IL-8 during flight^48^. A recently published study shows how the stress of microgravity can have a negative impact on the innate immune response of animals living in a symbiotic relationship^104^.

Henry et al. proposed two pathways in which the microbiome may affect host evolutionary potential. The first pattern proposed that microbial variation may shift the mean phenotype of the population, while the second pattern proposed that microbial variation may change host phenotypic variance. Both patterns may occur together, creating a framework in which genetic variation in the microbiome can extend the genetic repertoire of the host genome, influence host heritability, and thus impact host phenotypic evolution^105^.

### Vaccination

Enhanced pathogenicity, virulence, and drug resistance properties of microorganisms in space could pose a significant risk to the health of crewmembers during long-duration missions. Current approaches aim to identify the components of organisms that facilitate increased virulence in space, and then apply this information in targets for anti-microbial therapeutics, including vaccines^106^. Vaccines are an effective strategy for preventing viral diseases. Space-based platforms have led to a potential candidate vaccine for *Salmonella* and is currently in the early stages for review and development^107^. Moreover, space research aims to improve on existing vaccines, such as *Streptococcus pneumonia*, a bacterium that causes life-threatening diseases like pneumonia, meningitis, and bacteremia^108^. The 1-year twin study performed a vaccination response experiment to compare the effect of influenza immunization in the spaceflight environment with that on Earth. The immune system in space responded appropriately to the flu vaccine in all flight phases and compared to the ground control twin. There were no significant differences in the percentage of CD4^+^ and CD8^+^ T cell receptor sequences inflight compared to preflight and post-flight responses^20^. Astronaut vaccination proves to be a promising method for reducing space-induced infectious diseases, however, extensive research is required to guide astronauts during longer stays in space and destinations farther away from Earth.

## Evolution

### An evolutionary perspective

Survival of the fittest, a concept from the 19th century, describes how organisms that are best adjusted to their environment are more successful in survival and reproduction^109^. Research in space life sciences mainly focuses on understanding the physiological and psychological response of the human body to the space environment. However, in an evolutionary context we must consider how these changes impact human health and consequently the safety and survival of astronauts, and which adaptations will be naturally selected by this extreme environment. Human adaptations to spaceflight are generally denoted as maladaptations as they deviate from responses shaped by natural selection in terrestrial environmental conditions (i.e., weakening of host defense mechanisms, muscle wasting, and bone resorption). However, these adaptations are physiological responses to a new, extreme environment that is fundamentally different from our terrestrial world (see Box 1)^110^. For example, it is known that skeletal health and physical activity strongly influence the human immune system on Earth. Microgravity induces osteoporotic processes, with a bone mineral density loss of 1–2% per month spend in space. This adaptation is fundamentally different when compared to low-weight-bearing athletes on Earth. Swimmers’ bone density and structure showed adaptations to changes in gravity. They presented lower bone mineral density compared to high-impact athletes and sedentary controls, but demonstrated a higher bone turnover compared to controls, resulting in a different structure that was more resistant to fracture indexes^111^.

### Host-pathogen co-evolution

The rate of evolution is typically defined as the number of generations needed for an initially random population to achieve a given goal. Natural evolution occurs in temporally and spatially varying environments, the more complex the changes, the more dramatic the speedup^112^. The space environment therefore will evolve systems much faster. The host-pathogen relationship is an interesting example. Spaceflight is known to enhance microbial growth rates, antibiotic resistance, microbial invasion of host tissue, virulence, and genetic changes within the microbe^74–77^. The human microbiome on the other hand demonstrated reduced diversity after spaceflight, which can weaken the immune system^89,92–94^. These two intimately linked entities might be able to evolve in response to the space environment but might do so at two very different rates (Fig. 3c). On the evolutionary timescale, microbes tend to evolve faster due to shorter generation times and often stronger selection^113^. The evolutionary arms race between predator and prey, illustrated by the ‘Red Queen’ metaphor often refers to host-pathogen co-evolution, wherein both pathogen and host need to constantly invent new infection and protection measures to survive. Experimental evidence already showed the survivability of several microorganisms such as bacteria and spores to the space environment^114–116^. Maintaining a balanced host-microbiome relationship poses a major health challenge for astronauts.

### Small population size

We must consider that the success or failure of a variation will not be known until after it emerges^110^. As of February 2024, 681 people have reached the altitude of space according to the United States Air Force definition^117^. Agent-based modeling was used to simulate small-scale communities (i.e., human settlement) on Mars, drawing on high-performance teams in isolated and high-stress environments (ex. submarines, Artic exploration, war). The goal was to determine a minimum initial population which was to maintain or bounce back quickly (within 1.5 years) to a stable colony size equal to or greater than 10 for all 28 years^118^. An initial population size of 22 was the minimum required to maintain a viable colony size. In an evolutionary context, we deal with several small population size effects, including (1) genetic drift that describes random changes in gene frequencies, independent of mutation and natural selection^119^, (2) the founder principle that describes high frequencies of a specific genetic trait from a common ancestor and^120^, (3) the bottleneck effect that describes a dramatic reduction in genetic diversity of a species induced by catastrophic events^119^. All these effects will be of importance for space evolution, highlighting the importance of genetic variability. Another important factor we must consider is the immune variability between healthy individuals. This immune heterogeneity as reflected in their immunotypes, is a poor predictor of immune responses^121^.

## Spaceflight preparation

### Immune development

**Fig. 4:**
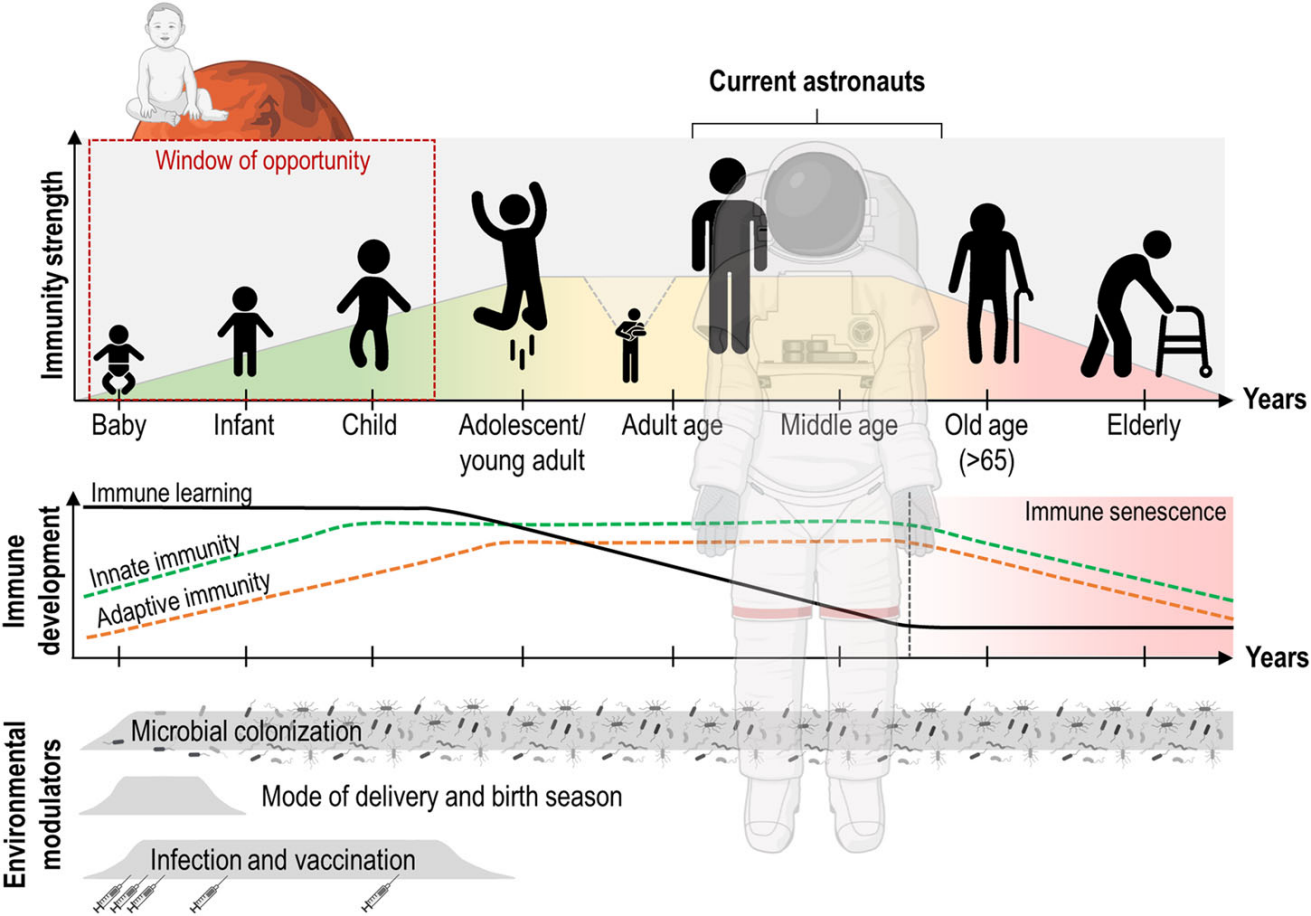
Development of the human immune system throughout his lifespan on Earth. The strength of the immune system (both innate and adaptive) builds up in the first years of life. However, its ability to learn decreases from early adulthood, before immune senescence begins around the age around 65. The astronauts recruited so far were all middle-aged, when the immunological learning capacity was already lower. The window of opportunity is a period in which microbial factors have a strong impact on the development of immune responses.

Newborns, in particular premature babies, have an impaired innate immunity, weak Th1 and antibody responses that make them more susceptible to bacterial and viral infections, resulting in high mortality rates observed in conditions of increased pathogen exposure. The immune system gradually matures during childhood (Fig. 4). Risks of infections slowly reduce due to vaccinations which stimulate protective immune responses. Children may still acquire bacterial, viral, and fungal infections that need to be fought off, adding to their immunological memory. Immunological memory persists into old age but may eventually fade. Over time, protection provided by immune responses increases, and young adults suffer fewer infections^122^. During pregnancy, the mothers’ immune system undergoes several changes to undermine the rejection of the semi-allogeneic graft. These changes include local immune suppression at the site of implantation, mediated by NK cells, monocytes, and regulatory T (T_reg_) cells. T cell activation is suppressed, and a shift is observed from Th1 to Th2 cell responses^123–125^. This immune modulation, however necessary for the well-being of the fetus, makes pregnant women more susceptible to severe complications of influenza and other infections^126^. As age advances, the immune system undergoes profound remodeling. Age-related reshaping of naive T cell repertoire, with a reduction of naive CD8^+^ T subsets, and change of the T-cell phenotype towards differentiated memory T-cells, altogether leads to an age-related reduction of the T-cell pool. This leads to a lower vaccination efficiency, decreased immune surveillance and resistance to infectious diseases, increased onset of reactivation of latent viruses, auto-immune diseases, and cancer. For this reason, older adults (65 years and older) see a significant increase in the rate of morbidity and mortality^122,127^. Rubelt et al. define the age of 50 and beyond as the onset of immune senescence observed through an age-dependent reduction of class switch recombination ability, likely underlying the reduced efficacy of vaccination^128^.

### Early immune exposure

Immune development knows a critical period, the so-called ‘window of opportunity’, that ranges from prenatal life to age six (school age; Fig. 4)^122^. The hygiene hypothesis, formulated by Strachan in the late 1980s, proposes the fundamental idea that early childhood exposure to appropriate levels of microorganisms protects against immune deviation and allergic diseases by strengthening the immune system. The timing of exposure to pathogens and the ensuing immune response is important for immune development. Strachan found an inverse correlation between hay fever and the number of older siblings^129^. Critical environmental modulators of the young immune system are mode and season of delivery^130–132^, infections and vaccinations^133^. Ever since, various studies have linked the development of allergies and autoimmune diseases to limited childhood microbial exposure in more Western, industrialized countries. For instance, the ‘Alpine farm studies’ identified traditional farming characteristics such as the consumption of unprocessed farm milk and close contact with farm animals to be allergoprotective and associated with a higher microbial load^134^. After the ‘fall of the Iron Curtain’ in 1989 between Western and Eastern Germany, a higher prevalence of allergies was observed in children from Western Germany, despite higher levels of pollution by industrial emissions in Eastern Germany, concluding that other exposures than pollutants influence the development of atopic diseases^135^. Poor immuno-regulation causes chronic inflammatory diseases that are increasing in prevalence in urban communities in high-income countries^136^. These studies highlight the crucial role of a diverse and rich immune-stimulating microbial environment in early life, in the establishment of a competent, tolerogenic, and defensive immune system, later in life. However, the functioning of a person’s immune system is adapted to their immediate environment. It is not possible to maintain the functionality of the immune system if the environment changes drastically. Most likely, future space travelers will have a completely different immune response (system) that is highly adapted to the space environment, unless they are otherwise challenged by a variety of antigens.

### Lifestyle improvements

Spaceflight involves many lifestyle adaptations, such as changes in diet, reduced physical activity, and new sleeping habits that can disturb circadian rhythm. All these lifestyle adaptations indirectly affect the human immune function. Major physiological improvements in spaceflight-induced immune dysfunction seem to have initiated approximately 11 years ago, a period coinciding with improvements onboard the ISS. Findings report a reduction in previously reported plasma cytokine increases, improvements in T cell blastogenesis, improved mitogen-stimulated cytokine profiles, reduced salivary cortisol levels during flight, reduction in the reactivation of latent EBV and CMV, and a complete ablation in the reactivation of VZV. Authors associated these improvements with the evolution of the ISS as a vehicle (e.g., additional parts increased habitable volume), deployment, and evaluation of various biomedical countermeasures (e.g., dietary improvements, better exercise countermeasures, crew psychology support, frequent resupply, etc.)^29^. For instance, a study investigated the relation between physical activity pre-, in-, and postflight with latent viral reactivation. Crewmembers with high cardiorespiratory fitness (CRF) levels preflight had a 29% reduced risk of latent viral reactivation inflight. Latent viral reactivation rates were highest in crewmembers with low preflight CRF levels and high CRF-deconditioning levels on return to Earth. Higher preflight upper body muscular endurance had a 39% reduced risk of viral reactivation, longer time to viral reactivation, and lower peak viral DNA concentrations (EBV and VZV)^137^. Furthermore, adequate nutrition is essential for a functioning healthy immune system^138^. Active areas of research focus on nutritional countermeasures such as supplements or probiotic microbes to prevent or mitigate infection^14^. For instance, probiotics such as *Lactobacillus casei* strain Shirota showed improvements in innate immunity and increased the NK cell activity by enhancing IL-12 production by monocytes and macrophages^139^. Probiotics derived from *Akkermansia*, a bacterium linked to host metabolism and immune response, may reduce the risk of chronic inflammatory diseases^140^. Another study showed that *Faecalibacterium prausnitzii* has anti-inflammatory properties by increasing the production of IL-10 and TNF in the colon to improve intestinal disease^141^. Finally, probiotics can produce short-chain fatty acids, which have a crucial part in the regulation of the immune system. Probiotics promoting short-chain fatty acid formation may boost nutritional and metabolic resources as well as lymphocytes’ capacity to eliminated viruses and potentially reduce latent viral reactivations^142^.

### Adaption or countermeasures?

The interplay between natural selection, culture, and technology will be important in the context of human evolution to space. For instance, will humans adapt to microgravity, colonize other planets, and as a result adapt to partial gravity conditions, or will artificial gravity preclude humans from having to adapt at all? To date, there are big gaps in the research regarding long-term immunological adaptation to the space environment and how will it adapt on such a small timescale. While there are serious grounds for concern, there have been very few medical emergencies in astronauts exposed to the relatively short-duration space missions. Future exploration missions will take astronauts away from Earth, bringing new challenges regarding autonomous healthcare and long-term exposure to the space environment. This sudden transition to space is hard to envision without failure to adapt. Movement away from Earth needs the accompaniment of countermeasures to secure safety and well-being of the astronauts and to possibly counteract unforeseen obstacles.

Taking a closer look on possibilities to avoid such an adaptation, scientists have been exploring artificial gravity to keep astronauts operating in a normal Earth-like environment. Artificial gravity can be produced in a number of ways. Linear acceleration is achieved by accelerating the spacecraft continuously in a straight line. Objects inside will be forced in the opposite direction of that applied acceleration. Orbital adjustments, made routinely by the thrusters of a spacecraft, are an example of linear acceleration. The duration of this artificial gravity however is of short duration and therefore not feasible as a countermeasure for deep space exploration missions and human evolution into space. Centrifugal acceleration is achieved by rotating or spinning the aircraft around its own center of mass. For example, a given gravity level is generated as a function of angular velocity (rotation rate, rpm) and distance from the center of rotation (radius). Artificial gravity, although a “classical countermeasure” in the sense that it would stabilize dysregulated immune function, is far from being realized yet.

A number of other potential immunological countermeasures for deep space exploration should be mentioned^143^, those of which are within the immediate control of the astronauts.

Nutritional countermeasures to reduce nutrient deficiencies or insufficiencies known to have profound effects on immune function. Hypocaloric nutrition, observed in earlier space missions, was associated with increased inflammation and oxidative stress^144,145^. Various foods and supplements are proposed to maintain immune function on exploration missions, including protein- and/or amino acid-rich foods and/or supplements^146^; food products with anti-oxidant functions (such as vitamin E^147^) and diets rich in fruit and vegetables which contain micronutrients such as carotenoids, vitamin C and folate^148^. Nutrient-rich diets might benefit, in accordance, the composition and expression of the gut microbiome, highlighting the importance of a symbiotic relationship between humans and their microbiome. Introduction of probiotic microbes to the space food might prove a potential countermeasure to immune dysregulation^149,150^. Other countermeasures that are already in place and can benefit from optimizations include individualized exercise regimen, adequate sleep schedules, and psychological support—family communication.

Because of the small sample size and small population size for astronauts who will travel into space, we cannot rely on community-based immunological countermeasures, such as herd immunity. For this reason, alternatives to herd immunity should be explored. Specific immunological countermeasures that are an active field of research at present include vaccinations, pharmacological interventions, and potential inflight monitoring of immune parameters^151,152^.

## Conclusions

The evolutionary potential of the human immune system in space deals with many (yet unknown) obstacles. Such challenges include different pathogenic potential in microorganisms, altered immune response, low genetic variability, and large immune variability due to small population sizes to name a few. Reflecting on the evolutionary journey of immunity to a future in outer space reveals a crucial insight: the intricate and multifaceted actions and interactions within innate and adaptive immunity stem from a rigorous and enduring process of selection and deselection as described. This ongoing process has progressively enhanced our ability to discern between self and non-self, enabling an effective defense against pathogens^153^. To which degree gravity changes, radiation or a lowered oxygen is affecting remains open, especially since the immune system emerged from hypoxic states. Monitoring of immune functions will be critical in the future to value the effects of countermeasures as well as the effects of aggravations along the time of exposure to the space exposome^154^.

One hypothesis can be formulated based on current findings that may guide the evolution of the human immune system into space: Will the human immune system better adapt to the space environment when it is exposed at a younger age? Infancy is the most critical period for immune development, where environmental modulators play a key role in the fine tuning of the immune response. Returning astronauts showed improved NK cell function and lower levels of AMPs, demonstrating the immune’s ability to adapt upon re-exposure. Not surprisingly, similarities were observed between immune senescence – the decline of the immune system with age, and the astronaut’s immune response to space. These include a reduced ability to respond to antigens, low-grade inflammation, and reactivation of latent viruses. Research into the effects of microgravity on regenerative health, specifically immune senescence, is gaining momentum^155^. Reducing the average age of astronauts flying to space, re-exposure studies, and spaceflight exposure during immune development might provide valuable insights for future space missions. The impact of complementary environmental factors such as confinement, habitat atmosphere, or pathogen compositions in enclosed environments together with radiation effects will certainly be of impact on the immune performance and to be included in such perspective.

The human immune system emerged in response to environmental needs spanning over billions of years. The sudden jump from Earth to outer space and foreign planets is accompanied by many health hazards and immunological challenges. The immune’s adaptation in such short timescales can therefore not be without failure. The need for countermeasures (e.g., vaccinations, artificial gravity, and environmental decontamination) is ever so important in humankind’s journey away from Earth.
